# Whole exome sequencing reveals mutations in *NARS2* and *PARS2*, encoding the mitochondrial asparaginyl-tRNA synthetase and prolyl-tRNA synthetase, in patients with Alpers syndrome

**DOI:** 10.1002/mgg3.115

**Published:** 2014-10-23

**Authors:** Kalliopi Sofou, Gittan Kollberg, Maria Holmström, Marcela Dávila, Niklas Darin, Claes M Gustafsson, Elisabeth Holme, Anders Oldfors, Már Tulinius, Jorge Asin-Cayuela

**Affiliations:** 1 Department of Pediatrics, University of Gothenburg, The Queen Silvia's Children HospitalGothenburg, Sweden; 2 Department of Clinical Chemistry, University of Gothenburg, Sahlgrenska University HospitalGothenburg, Sweden; 3 Bioinformatics Core Facility, University of GothenburgGothenburg, Sweden; 4 Department of Medical Biochemistry and Cell Biology, University of GothenburgGothenburg, Sweden; 5 Department of Pathology, University of Gothenburg, Sahlgrenska University HospitalGothenburg, Sweden

**Keywords:** Alpers syndrome, *NARS2*, *PARS2*, whole exome sequencing

## Abstract

Alpers syndrome is a progressive neurodegenerative disorder that presents in infancy or early childhood and is characterized by diffuse degeneration of cerebral gray matter. While mutations in *POLG1*, the gene encoding the gamma subunit of the mitochondrial DNA polymerase, have been associated with Alpers syndrome with liver failure (Alpers–Huttenlocher syndrome), the genetic cause of Alpers syndrome in most patients remains unidentified. With whole exome sequencing we have identified mutations in *NARS2* and *PARS2*, the genes encoding the mitochondrial asparaginyl-and prolyl-tRNA synthetases, in two patients with Alpers syndrome. One of the patients was homozygous for a missense mutation (c.641C>T, p.P214L) in *NARS2*. The affected residue is predicted to be located in the stem of a loop that participates in dimer interaction. The other patient was compound heterozygous for a one base insertion (c.1130dupC, p.K378 fs*1) that creates a premature stop codon and a missense mutation (c.836C>T, p.S279L) located in a conserved motif of unknown function in *PARS2*. This report links for the first time mutations in these genes to human disease in general and to Alpers syndrome in particular.

## Introduction

Mitochondrial disorders affect the generation of cellular energy through oxidative phosphorylation (OXPHOS) (DiMauro et al. 2013). The assembly and function of the mitochondrial OXPHOS machinery relies on a large number of proteins, mainly encoded by not only the nuclear DNA but also the mitochondrial DNA. Genetic defects in some of these proteins give rise to a wide spectrum of disease phenotypes with different age of onset and course of disease. Among the early-onset mitochondrial disorders with central nervous system involvement, Alpers syndrome is one of the most common phenotypes (Darin et al. 2001).

Alpers syndrome is a progressive neurodegenerative disorder of infancy and early childhood, with characteristic diffuse degeneration of cerebral gray matter (Alpers 1931). Mutations in *POLG1* have been found to cause Alpers syndrome with hepatic dysfunction, a syndrome that is better known as Alpers–Huttenlocher (Huttenlocher et al. 1976; Kollberg et al. 2006). Besides the presence of hepatic dysfunction, often secondary to valproate treatment, which can result in liver failure, the patients with Alpers–Huttenlocher syndrome have a severe and rapidly progressive disease course, characterized by psychomotor regression, ataxia, refractory seizures, epilepsia partialis continua, convulsive status epilepticus, and stroke-like episodes (Sofou et al. 2012). This phenotype differs from Alpers syndrome not caused by mutations in *POLG1*. The latter presents often perinatally and exhibits a more protracted disease course. Patients with Alpers syndrome present with neonatal seizures, infantile spasms, or generalized seizures and develop spasticity and progressive microcephaly with accompanying severe mental retardation (Sofou et al. 2012). Liver dysfunction may also be present in Alpers syndrome, but is usually mild and sometimes spontaneously resolving.

The genetic etiology underlying Alpers syndrome is in most patients unknown. Recently, mutations in *FARS2*, a gene encoding the mitochondrial phenylalanyl-tRNA synthetase, have been identified in two Finnish patients with Alpers syndrome (Elo et al. 2012). This enzyme belongs to the class II aminoacyl-tRNA synthetase (mt-aaRS) family and is responsible for charging the mitochondrial tRNA^Phe^ with phenylalanine. This function, like that of all the other mitochondrial aminoacyl-tRNA synthetases, is essential for efficient mitochondrial protein synthesis (Suzuki and Nagao 2011).

In a previous study involving 19 patients, six of them had Alpers–Huttenlocher syndrome and all presented pathogenic mutations in *POLG1*. For the other 13, who presented with Alpers syndrome, the underlying genetic defect remained elusive (Sofou et al. 2012). Here we report new genetic data obtained by whole exome sequencing, concerning patients #13 and #15 from that previous study, who in addition to the encephalopathy developed renal dysfunction and cardiomyopathy, respectively. We have identified novel mutations in *NARS2* (OMIM: 612803) in patient #13 and *PARS2* (OMIM: 612036) in patient #15. These genes encode the mitochondrial asparaginyl-and prolyl-tRNA synthetases. This is, to the best of our knowledge, the first report linking mutations in these genes to human disease.

## Patients and Methods

The parents of both patients gave their written consent to carry out the investigations reported.

### Patient description

#### Patient I (#13 in Sofou et al. 2012)

This boy was the second child to healthy, nonconsanguineous parents of Swedish descent. His older sister was healthy. The mother's pregnancy was complicated by hyperemesis gravidarum. The patient was born with elective cesarean section at 38 weeks of gestation. At birth, his weight was 2.280 g (−2.5 SD), his length was 48 cm (−1 SD), while his head circumference was around the mean (33.5 cm). The early infantile period was characterized by inconsolable crying, tendency to opisthotonus posturing, and delayed head control. The patient's psychomotor development reached its maximum level at the age of 6 months, which corresponded to a developmental age of 3 months. Then, the patient showed the first signs of psychomotor regression, with loss of previously acquired motor skills, and feeding difficulties. The patient was admitted to hospital at the age of 6.5 months because of a febrile illness, during which the patient developed poor eye contact and hypotonia with complete head lag. CT scan of the brain displayed cortical atrophy. At 7 months of age the patient developed generalized seizures of multiple types, including myoclonic, tonic, and atypical absence seizures. Repeated electroencephalography (EEG) tests demonstrated bilateral synchronous spikes and polyspikes, mainly in the posterior regions of the hemispheres, with generally depressed background activity. Lactate levels were elevated in blood up to 6.1 mmol/L (reference value <1.7 mmol/L) and in CSF up to 2.6 mmol/L (reference value <1.7 mmol/L). A muscle biopsy was performed at 7.5 months of age. The biochemical, morphological, and histopathological findings are presented in Biochemical and Morphological Investigations section. Ophthalmological examination at 2.2 years of age revealed optic atrophy and nystagmus. The patient later developed cortical visual impairment leading to blindness. MRI of the brain performed at 3.5 years of age showed profound supratentorial atrophy of the cerebral cortex, complete agenesis of the corpus callosum, and hypomyelination of the white matter. Because of feeding difficulties and persistent vomiting associated with gastro-esophageal reflux, the patient underwent laparoscopic fundoplication with gastrostomy at the age of 4.5 years. In the following years, the patient developed progressive microcephaly, severe mental retardation, spastic tetraparesis, and scoliosis. During infections, the patient was repeatedly found to have hypochloremic metabolic alkalosis (pH: 7.55, reference range: 7.38–7.46, serum bicarbonate: 36 mmol/L, reference range: 21–27 mmol/L, serum chloride: 84 mmol/L, reference range: 96–106 mmol/L) and hyponatremia (serum sodium: 127 mmol/L, reference range: 136–144 mmol/L). He was admitted to the intensive care unit at 9 years of age because of acute dyspnea due to pulmonary edema. Upon admission, the patient had metabolic alkalosis (pH: 7.60, serum bicarbonate: 34 mmol/L), hypoalbuminemia (serum albumin: 17 g/L, reference range: 35–50 g/L), hypokalemia (serum potassium: 1.9 mmol/L, reference range: 3.5–5.0 mmol/L), hyponatremia (serum sodium: 133 mmol/L), and hypocalcemia (serum calcium ion: 0.4 mmol/L, reference range: 1.20–1.38 mmol/L). The patient had also glycosuria, proteinuria, and increased salt excretion in urine. The liver transaminases were slightly elevated (serum alanine aminotransferase: 1.8 *μ*kat/L, reference value: <0.70 *μ*kat/L and serum aspartate aminotransferase: 2.0 *μ*kat/L, reference value: <0.33 *μ*kat/L). The cardiac function was normal. The patient's serum creatinine level increased up to 138 *μ*mol/L in the following days (reference range: 20–60 *μ*mol/L). During the ensuing weeks, the serum creatinine levels normalized, but glycosuria and increased urinary salt excretion persisted and the patient required lifetime supplementation with calcium and potassium. During hospitalization and for a period after discharge, his epileptic seizures worsened and were resistant to combination therapy with valproate and benzodiazepines. The patient showed a slowly progressive clinical course without signs of liver failure, and died at the age of 16 years. The postmortem findings are summarized in Biochemical and Morphological Investigations section.

#### Patient II (#15 in Sofou et al. 2012)

This boy was the only child to healthy, unrelated parents of Swedish descent. He was born after an uncomplicated pregnancy at 42 weeks of gestation. At birth his weight was 4.880 g (+2 SD), his length was 58 cm (+3 SD), and his head circumference was 36 cm (+1 SD). The neonatal period was normal. He was admitted to hospital at 2.5 months of age because of feeding difficulties following an upper respiratory tract infection. During hospitalization, he developed focal seizures with secondary generalization and signs of psychomotor regression. A brain CT revealed generalized cortical atrophy. Lactate levels were elevated both in blood up to 3.7 mmol/L (reference value: <1.7 mmol/L) and in CSF up to 4.2 mmol/L (reference value: <1.7 mmol/L). Repeated EEG showed bilateral spikes and polyspikes in combination with low-frequency activity. Besides generalized seizures, the patient also developed infantile spasms. Hypsarrhythmia was evident on EEG during a very short period. The patient received antiepileptic treatment with phenobarbital and clonazepam. He underwent muscle biopsy at 4 months of age. The biochemical, morphological, and histopathological findings are presented in Biochemical and Morphological Investigations section. A gastrostomy was performed the same month, due to the patient's severe feeding and swallowing difficulties. In the following months, the clinical course was characterized by generalized hypotonia, dystonia, progressive microcephaly, severe mental retardation, and cortical visual impairment. Another CT of the brain performed at 10 months of age showed progression of the cerebral cortical atrophy. The patient suffered from repeated respiratory tract infections and gastro-esophageal reflux. No signs of liver dysfunction were present. By the age of 2 years, the patient's weight was at the normal mean for age, but his height was +5 SD and his head circumference was less than −3 SD. The patient was admitted to the intensive care unit at 2 years of age because of a severe episode of persistent vomiting complicated by circulatory collapse. A heart ultrasound showed dilated cardiomyopathy and left ventricular hypertrophy. At 2 years and 2 months of age the patient died of heart failure. The postmortem findings are summarized in Biochemical and Morphological Investigations section.

### Biochemical and morphological studies

Mitochondria were isolated from fresh biopsy samples obtained from the vastus lateralis muscle as described previously (Bookelman et al. 1978). Polarographic measurements on fresh mitochondria and respiratory chain enzyme activities on frozen mitochondrial preparations were performed as described previously (Tulinius et al. 1991). Parts of the biopsy material were frozen in propane chilled in liquid nitrogen for histochemical investigations or fixed in glutaraldehyde for electron microscopy as described previously (Tulinius et al. 1991). Postmortem investigations were performed in both patients. Specimens from various parts of the brain as well as other organs were fixed in paraformaldehyde and embedded in paraffin for morphological examinations.

### Western blotting analysis

Western blotting was performed on whole cell homogenate from fibroblast from patients I and II and several age, gender, and passage number matched controls following standard procedures. Polyclonal rabbit antibodies against asparaginyl-tRNA synthetase and prolyl-tRNA synthetase were purchased from Novus Biologicals (Littleton, CO), while the antibody against glyceraldehyde-3-phosphate dehydrogenase (GAPDH) was obtained from Santa Cruz Biotechnology (Dallas, TX).

### Whole exome sequencing and data analysis

Whole exome sequencing (WES) was carried out by BGI Hong Kong Co., Limited (Hong Kong, China), using Illumina HiSeq 2000 high-throughput sequencing technology. Agilent (Santa Clara, CA) SureSelect Human All Exon 51M kit was used for exome capture.

Quality assessment of the sequence reads was performed by generating QC statistics with FastQC (http://www.bioinformatics.bbsrc.ac.uk/projects/fastqc). Read alignment to the reference human genome (hg19, UCSC assembly, February 2009) was done using BWA (Burrows–Wheeler alignment) with default parameters (Li and Durbin 2009). A summary of the sequencing data is shown in Table1. After removal of PCR duplicates using Picard tools (http://picard.sourceforge.net) and file conversion using SAMtools (Li et al. 2009) quality score recalibration, indel realignment and variant calling were performed with the Genome Analysis Toolkit package (McKenna et al. 2010). For the identification of potentially pathogenic variants, we used Ingenuity ® Variant Analysis ™ software (http://www.ingenuity.com/variants), from Ingenuity Systems. All mutations identified by WES were verified by Sanger sequencing using primers amplifying the exons harboring the mutations in *NARS2* and *PARS2*. Sequencing analysis was performed using an ABI PRISM ® 3100 Genetic analyzer and the BigDye Terminator v.1.1 Cycle Sequencing Kit (Applied Biosystems Foster City, CA). Primer sequences and PCR conditions are available upon request.

**Table 1:** Overall results of whole exome sequencing

Sample	Raw reads	Mapped reads	SNPs	Indels
Patient I	43,310,060	43,171,550	117,547	7084
Patient II	45,973,822	45,850,330	121,578	7551

## Results

### Biochemical and morphological investigations

Muscle mitochondria from both patients showed low levels of oxygen consumption in the presence of substrates for complex I and complex IV. Patient I showed a general decrease in respiratory chain enzyme activities, whereas in patient II the decrease was confined to complex I and complex IV (Table2).

**Table 2:** Respiratory chain complex activities in Patients I and II

			Reference interval	
	Patient I	Patient II	Mean	Range
Polarography	nmol/min per mg protein			
Substrate				
Pyruvate + malate	51	52	98	86–116
Glutamate + malate	50	59	107	86–125
Palmitoylcarnitine + malate	NA	42	63	30–84
Succinate + rotenone	90	94	99	76–117
Ascorbate + TMPD	155	160	251	207–302
Enzyme activities	*μ*mol/min per mg protein			
NADH/ferricyanide reductase	4.0	3.5	6.7	4.5–8.6
Succinate/cytochrome *c* reductase	0.11	0.19	0.30	0.16–0.40
Citrate synthase	2.2	1.5	2.4	2.0–3.5
	L/min per mg protein			
Cytochrome *c* oxidase	5.0	5.6	11	6.1–15

NA, not analyzed; TPMD, *N,N,N′,N′*-Tetramethyl-*p*-phenylenediamine.

**Figure 1:**
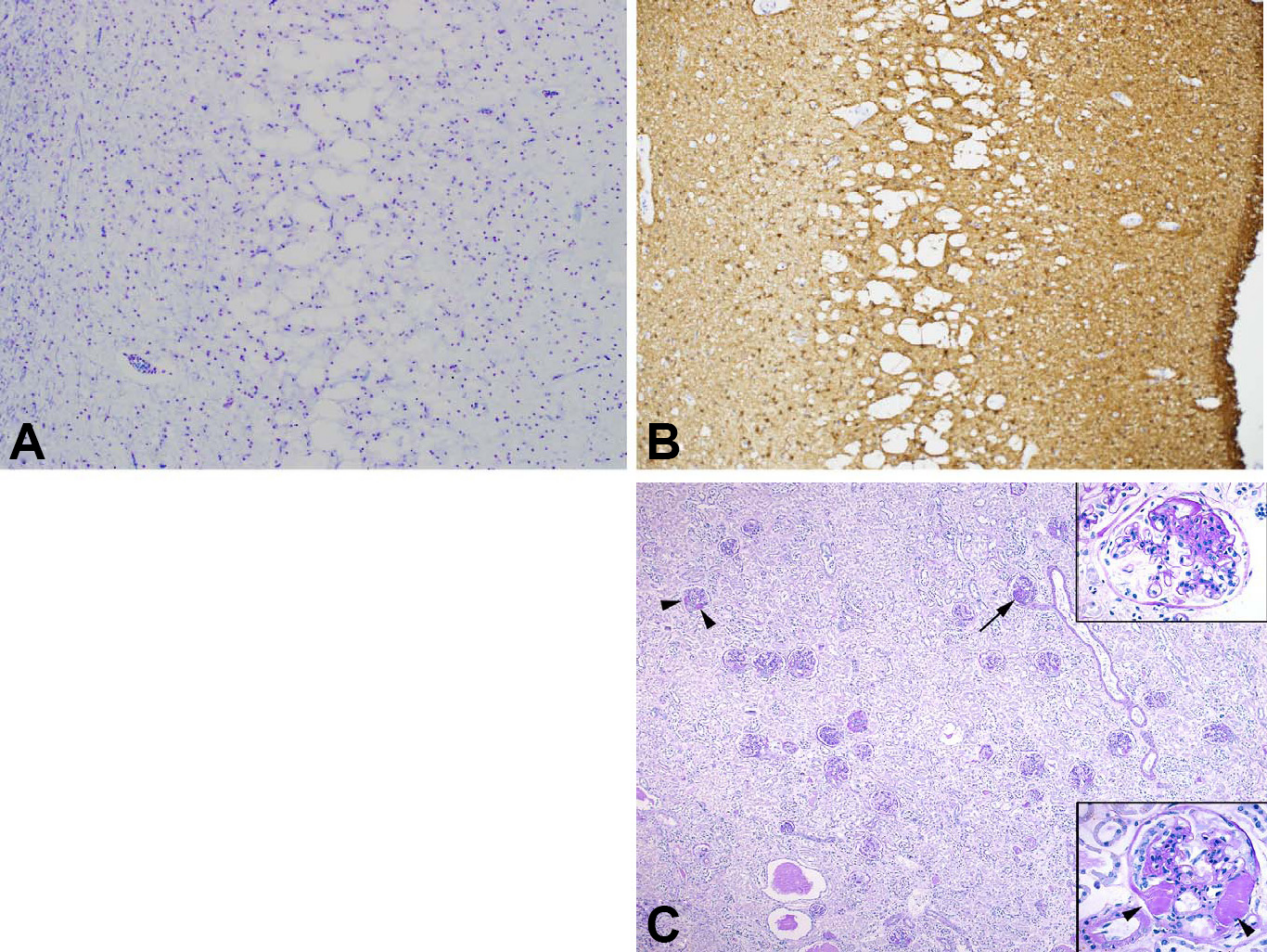
(A, B) Degeneration of the cerebral cortex with vacuolization in the middle layers, marked loss of nerve cells and prominent gliosis in patient I. The images illustrate the entire cortex from the white matter (left) to the pial surface (right). (A) Luxol fast blue/Cresyl violet, 50×. (B) Immunostaining of glial acidic fibrillary protein (GFAP, 50×). (C) Representative kidney section with occasional focal segmental glomerulosclerosis (FSGS, arrow and upper inset). There was a prominent nodular hyalinosis with a perihilar distribution in several glomeruli (arrowhead and lower inset). The parenchyma was fairly normal except for occasional small cysts and autolysis (periodic acid and Schiff (PAS) 15×; insets 100×). Courtesy Johan Mölne.

Skeletal muscle biopsy in patient I demonstrated increased fiber caliber variation, but no mitochondrial proliferation or enzyme deficiency as revealed by enzyme histochemistry. Electron microscopy revealed structurally abnormal mitochondria. The postmortem investigation in patient I revealed generalized severe atrophy of the brain, which weighed only 439 g. The cerebral cortex showed widespread degeneration and vacuolization with prominent gliosis and very few remaining nerve cells (Fig.1A and B). The cerebral white matter was reduced in volume and showed some gliosis but these changes appeared to be secondary to the cortical degeneration. There was no spongiosis of the white matter. The thalamus and basal ganglia showed degeneration with loss of nerve cells but less marked than in the cortex. The cerebellum showed marked cortical atrophy with severe loss of nerve cells, spongiosis, and gliosis. The cerebellar white matter was reduced in volume but without spongiosis. Other pathological findings were fatty change in the liver with a centrilobular distribution, bronchitis, and bronchopneumonia. Kidney tissue sections showed normal architecture with no signs of dysplasia. Occasional small cysts were found, but no cystic disease. There was an increased number of sclerotic glomeruli and occasional focal segmental sclerosis (FSGS). Prominent perihilar hyaline nodules were observed, but only minor focal hyalinosis in arterioli (Fig.1C). There was no increased interstitial fibrosis and only a few atrophic tubules. The heart was normal by macroscopic and microscopic investigation.

**Figure 2:**
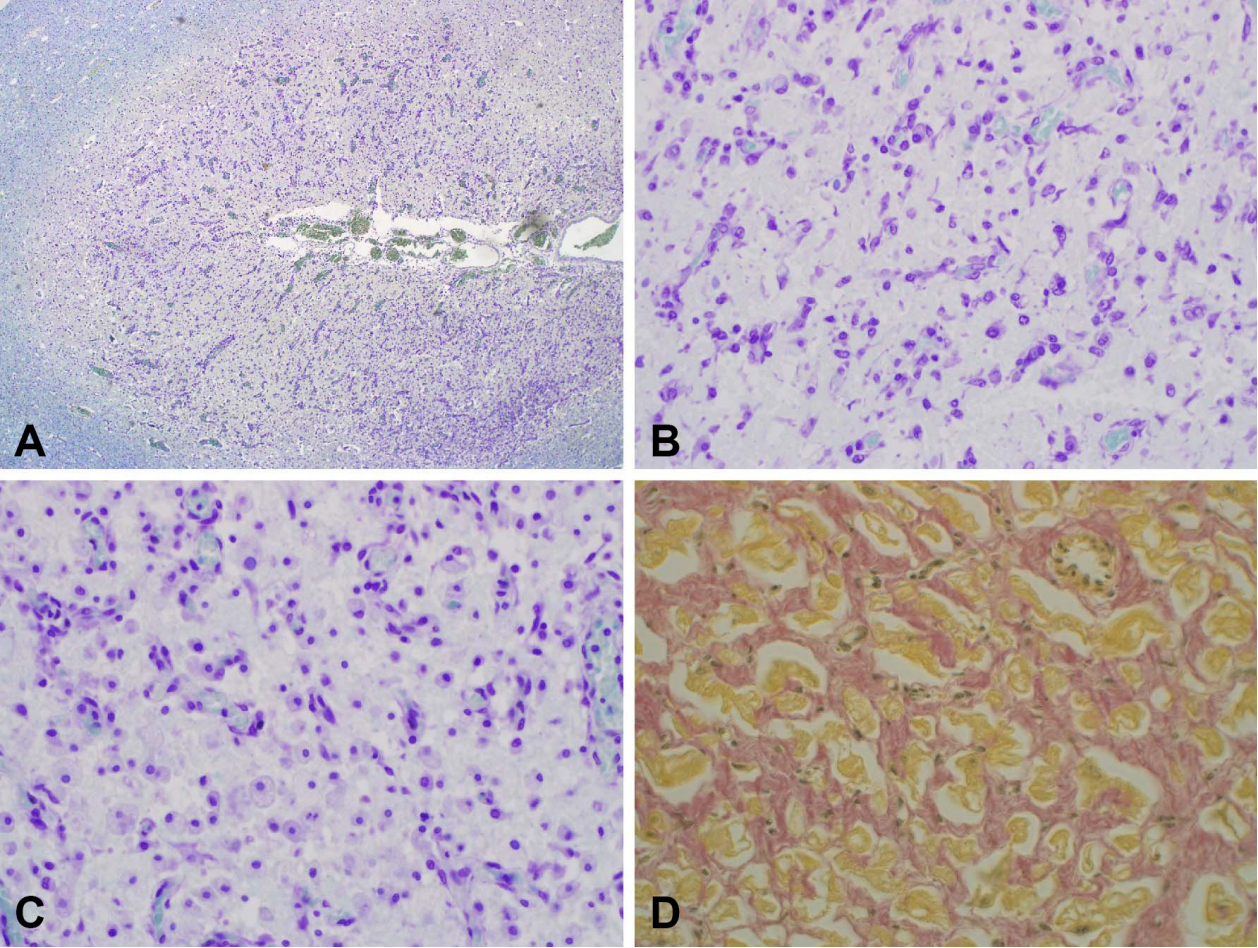
(A, B) Degeneration with loss of nerve cells, gliosis, and capillary proliferation in the brain cortex of patient II. The images illustrate the cortex adjacent to a sulcus of the brain cortex (A) overview 15×. (B) Detail demonstrating extreme loss of nerve cells and glial cell proliferation (Luxol fast blue/Cresyl violet, 150×). (C) Necrosis of the putamen in patient II with loss of nerve cells and infiltration of macrophages, many of them exhibiting features of foam cells (Luxol fast blue/Cresyl violet, 150×). (D) Myocardium of patient II demonstrating marked interstitial fibrosis (hematoxylin/van Gieson, 150×).

Skeletal muscle biopsy in patient II was essentially normal. The postmortem investigation in patient II revealed atrophy of the brain that weighed 834 g. The cerebral cortex demonstrated widespread lesions in all parts with atrophy or laminar necrosis with gliosis and capillary proliferation (Fig.2A and B). There were also parts of the cortex that revealed normal structure without evidence of nerve cell loss or gliosis. The basal ganglia showed necrosis affecting particularly the striatum (Fig.2C) but sparing globus pallidus. The cerebral white matter was essentially spared but revealed some minor spongiotic change. The heart was markedly enlarged with dilation of the ventricles and also increased thickness of the ventricle walls. It weighed 200 g with macroscopic and microscopic interstitial fibrosis of variable degree (Fig.2D). The liver was severely congested. The kidneys demonstrated no apparent macroscopic or microscopic pathological changes.

### Genetic findings

**Figure 3:**
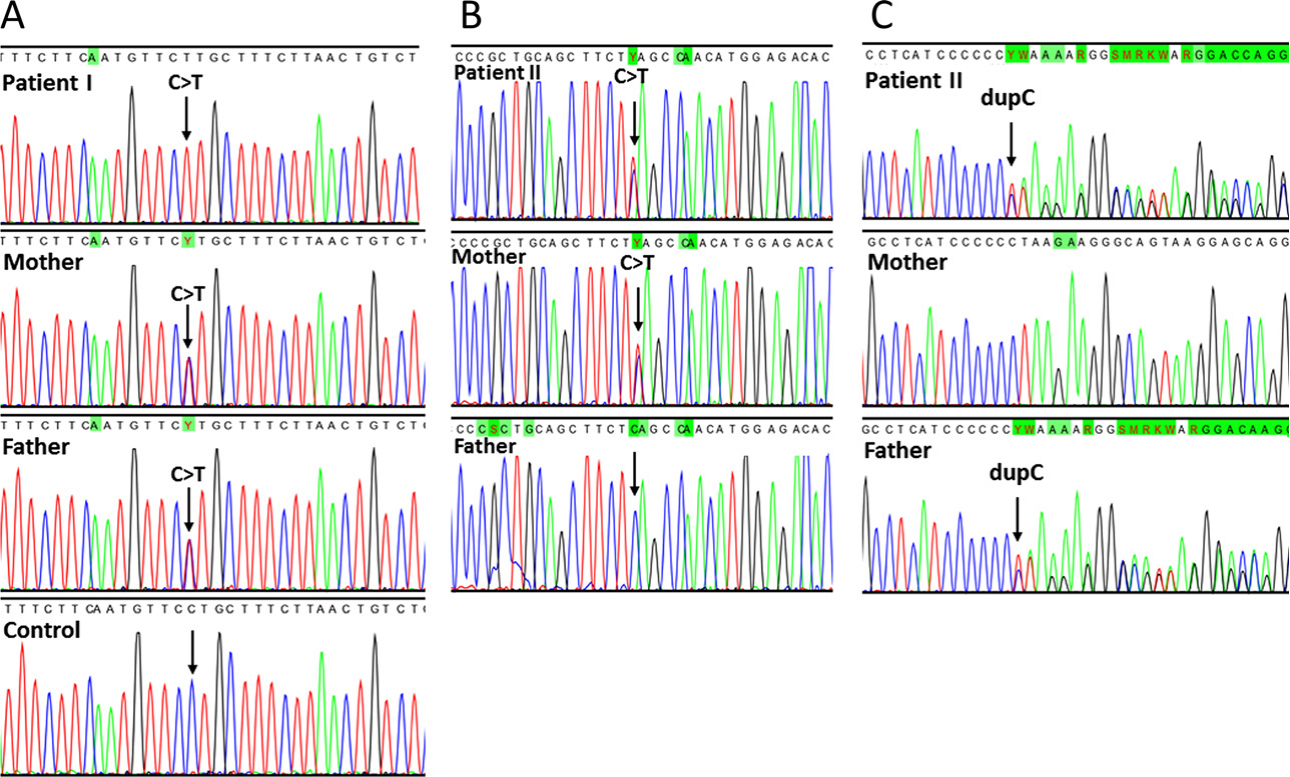
Sequencing chromatograms confirming the whole exome sequencing findings. (A) The arrow shows the homozygous c.641 C>T transition in *NARS2* in patient I. (B) Patient II presented a heterozygous c.836 C>T transition inherited from the mother and a heterozygous c.1130dupC mutation from the father in *PARS2*.

After variant calling and quality control, the search for pathogenic mutations that could explain the phenotype was carried out by sequentially applying four filters as shown in Table3. The first filter selected variants present in nuclear genes encoding mitochondrial proteins according to MitoCarta (Pagliarini et al. 2008). The second filter retained variants that presented an allele frequency <3% in either the 1000 genomes project (Abecasis et al. 2012), the public Complete Genomics genomes (Drmanac et al. 2010), or all NHLBI ESP (Exome Variant Server, NHLBI GO Exome Sequencing Project (ESP), Seattle, WA (http://evs.gs.washington.edu/EVS/ [December 2013]). The third filter excluded variants that were not predicted to be deleterious according to default IVA criteria and the fourth and last filter excluded variants that did not conform to a recessive pattern of inheritance. The entire mitochondrial DNA of both patients was sequenced previously (Sofou et al. 2012) and therefore it was not included in the analysis. This strategy rendered only one candidate gene for each patient. Patient I presented a homozygous mutation (c.641C>T, p.P214L, chr11:78239936) in *NARS2*, while patient II presented compound heterozygosity for two mutations (c.1130dupC, p.K378 fs*1, chr1:55223704/c.836C>T, p.S279L, chr1:55223999) in *PARS2*. The results were verified by Sanger sequencing and targeted sequencing on the parents showed the expected segregation pattern (Fig.3). The mutations identified were not present in HBVDB, a database containing sequencing data from 200 Danish and 249 Swedish individuals (Human Background Variation DataBase, http://neotek.scilifelab.se/hbvdb/). *NARS2* and *PARS2* are genes predicted to encode the mitochondrial asparaginyl-and prolyl-tRNA synthetases, respectively. Both enzymes belong to the aminoacyl-tRNA synthetase (mt-aaRS) family and are responsible for charging mitochondrial tRNAs with their cognate amino acids.

**Table 3:** Identification of candidate genes by whole exome sequencing

	Patient I		Patient II		
	Variants	Genes	Variants	Genes
Total	124,631	15,978	129,098	16,015
Genes encoding mitochondrial protein	1698	671	1882	681
Allele frequency <3%	98	94	100	86
Predicted deleterious	32	27	18	18
Recessive pattern of inheritance	1	1	2	1

**Figure 4:**
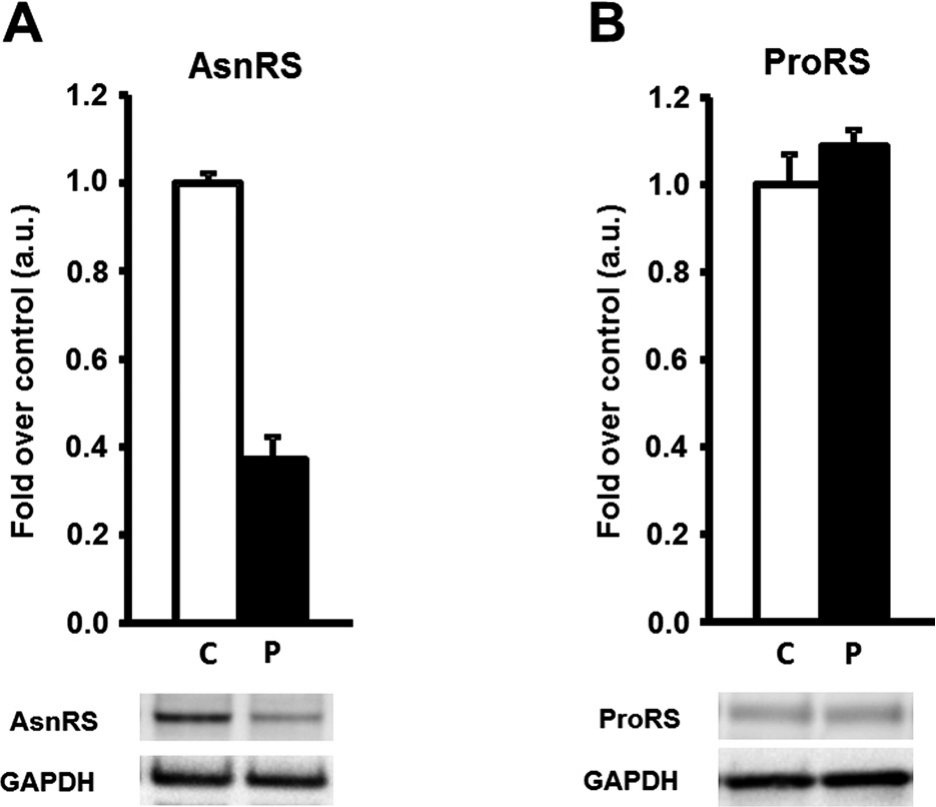
Asparaginyl-tRNA synthetase (AsnRS) and prolyl-tRNA synthetase (ProRS) abundance in control and patients cultured fibroblasts assessed by Western blotting. AsnRS (A) and ProRS (B) protein abundance in controls versus patient. Data are normalized to glyceraldehyde-3-phosphate dehydrogenase (GAPDH) abundance and presented as mean ± SE. Controls *n* = 3–4, with 1–5 independent repeats each. Patient *n* = 1, with four independent repeats each.

As a first approach to assess the functional consequences of the mutations identified we performed Western blotting analysis on cell extracts from cultured fibroblasts from both patients. As shown in Figure4, patient I showed a clear decrease in the steady state levels of asparaginyl-tRNA synthetase (37% of control levels on average). However, the levels of prolyl-tRNA synthetase in patient II did not differ significantly from control levels when normalized to GAPDH.

## Discussion

The genetic cause of Alpers syndrome not associated with *POLG1* mutations has remained obscure. A recent report identified mutations in *FARS2*, the gene encoding the mitochondrial phenylalanine-tRNA synthetase, in two patients with Alpers syndrome (Elo et al. 2012). Up till now, 11 mt-aaRS have been linked to human disease (Konovalova and Tyynismaa 2013). Despite the fact that all of them ultimately affect mitochondrial protein synthesis in all tissues, the clinical expression of these genetic defects is very heterogeneous. Brain is the most affected organ, but heart, cochlea, ovary, kidney, and muscle are specifically affected organs for some of these defects (Konovalova and Tyynismaa 2013, and references therein). This clinical heterogeneity has been further evidenced by the report of one patient with pathogenic mutations in *FARS2* presenting with infantile-onset epilepsy and complex IV deficiency who did not show any signs of cerebral gray matter degeneration. Instead, the patient presented structural brain abnormalities involving the corpus callosum and subcortical white matter lesions (Almalki et al. 2014).

The two patients that we have investigated showed diffuse degeneration of cerebral gray matter which is characteristic of Alpers syndrome, but in addition both presented with involvement of other organs. Patient I, harboring a homozygous mutation in *NARS2*, developed renal dysfunction with persistent glycosuria and abnormal urinary salt excretion due to proximal and distal tubulopathy as well as hypochloremic metabolic alkalosis. The patient had focal segmental glomerulosclerosis without developing nephritic syndrome. Renal disease has also been observed in patients with mutations in *SARS2*, the gene encoding the mitochondrial seryl-tRNA synthetase. These patients show infantile onset of a multiorgan disease known as HUPRA syndrome, with renal disease characterized by distal tubular dysfunction and renal failure (Belostotsky et al. 2011). The renal disease of patient I was not fully investigated, so we cannot judge whether the disease is of the same kind as in *SARS2* deficiency or not. Patient II, who was compound heterozygous for mutations in *PARS2*, developed dilated and hypertrophic cardiomyopathy at 2 years of age. Mutations in *AARS2*, the gene encoding the mitochondrial alanyl-tRNA synthetase, have also been associated with infantile mitochondrial cardiomyopathy (Gotz et al. 2011). Intriguingly, patient II had also macrosomy that reached +5 SD at 2 years of age, which was significantly longer than the parental target height (∼ +2 SD). This finding is quite unique among patients with mitochondrial disorders.

The identification of human diseases coupled to genes encoding mt-aaRS is very recent and for most of them only a few cases have been identified. It remains to be seen whether the phenotypes observed so far are typical of each defect or, as in the case of *FARS2*, heterogeneity is present even between patients harboring mutations in the same genes. Consequently, more patients need to be identified to determine whether gray matter degeneration with either tubulopathy or cardiomyopathy are hallmarks of defects in *NARS2* and *PARS2*, respectively, or just examples of a wider clinical spectrum.

Despite their phenotypic heterogeneity, one aspect that all the defects in genes encoding mt-aaRS share is the fact that complete loss of function of the respective enzymes is not compatible with life (Konovalova and Tyynismaa 2013). Consequently, pathogenic mutations located in these genes must allow for some residual activity.

Patient I presented a homozygous mutation in *NARS2*. This gene encodes 2 isoforms, named isoform 1 (NM_001243251.1) and isoform 2 (NM_024678.5). The mutation is located in the 3′-UTR region of isoform 2 (c.-41C>T), whereas in isoform 1 it causes a change in amino acid affecting a conserved residue (c.641C>T, p.P214L). This mutation is present neither in the list of dbSNPs (build 135) nor in any of the NHLBI ESP or HBVDB databases and is predicted to be damaging/probably damaging by SIFT/PolyPhen-2.

**Figure 5:**
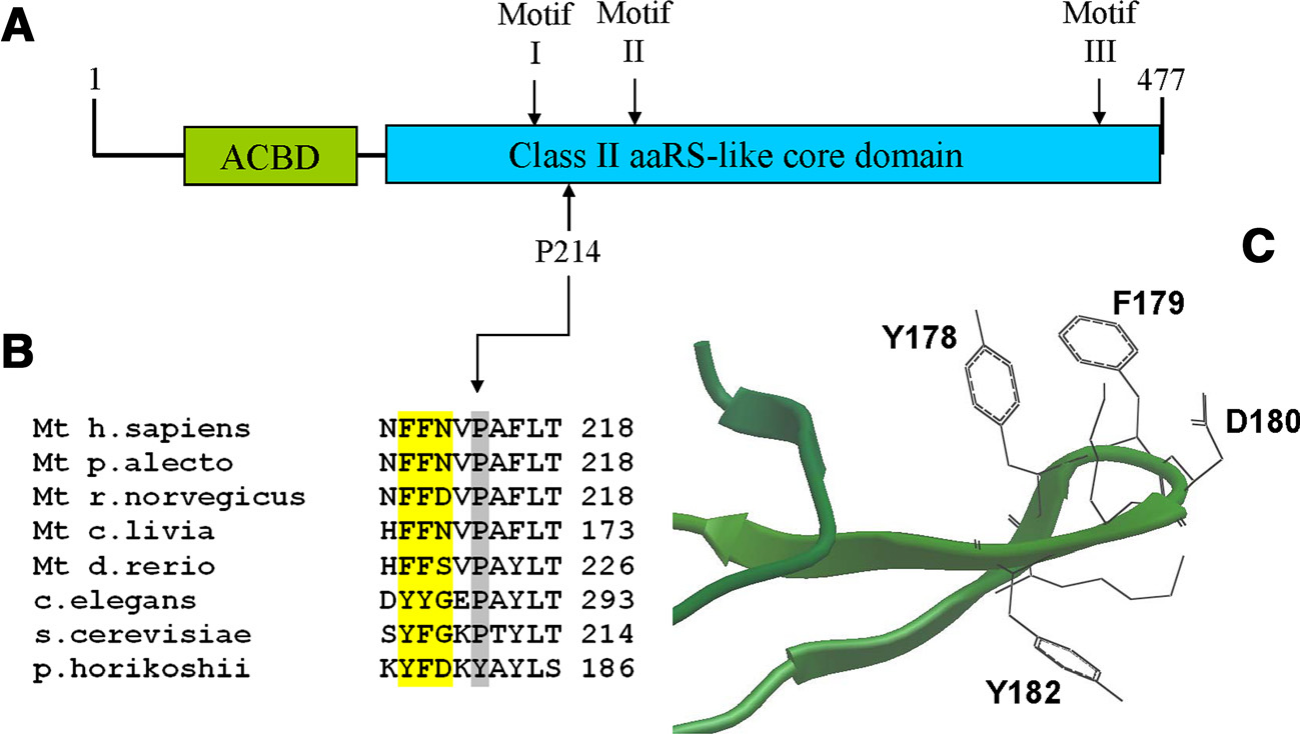
(A) Primary structure and principal domains of human *NARS2*. ACDB: anticodon-binding domain. (B) The mutated proline at position 214 is located in close vicinity to three residues that participate in monomer–monomer interaction (highlighted in yellow). (C) Detail of the crystal structure of asparaginyl-tRNA synthetase of *P. horokoshii* showing the loop from where the side chains of the three amino acids protrude. The mutated proline in the human protein is located at the step of the loop, in the position occupied by Y182 in *P. horokoshii*. The image was obtained with the Molsoft MolBrowser 3.7-2a browser.

Isoform 1 of mitochondrial asparaginyl-tRNA synthetase is predicted by CD-search (Marchler-Bauer and Bryant 2004) to contain an anticodon-binding domain at the N-terminus and a class II aaRS-like core domain, which stretches 2/3 of the total length of the protein including its C-terminus (Fig.5A). This domain contains the three motifs found in other class II aaRS. Most class II aaRS are homodimers, and motif 1 is involved in dimer interface formation, while motifs 2 and 3 constitute the active site. Motif 3 binds ATP, and motif 2 participates in coupling ATP, amino acid, and 3′-tRNA binding (Perona and Hadd 2012). On the basis of the crystal structure of the protein homolog in *Pyrococcus horikoshii* (Iwasaki et al. 2006), we can predict that the affected proline is located in the stem of a loop from which two very conserved aromatic residues (tyrosine and/or phenylalanine) protrude and physically participate in monomer–monomer interaction (Fig.5C). Proline plays a very important role in the secondary structure of proteins due to its conformational rigidity. The structural change caused by the mutation in this loop can therefore be expected to affect the activity of the protein by destabilizing the interaction between the two monomers. That kind of structural modification could leave some residual activity, which as mentioned above is expected in pathogenic mutations affecting mt-aaRS coding genes. Isoform 2 is a much smaller protein compared to isoform 1 (250 amino acids vs. 477), lacking the first 227 residues located at the N-terminus. Consequently, isoform 2 lacks the entire anticodon-binding domain, a fraction of the class II aaRS-like core domain, including motif I and most likely even the mitochondrial targeting sequencing, which in most mitochondrial proteins is located at the N-terminus. It is therefore extremely unlikely that isoform 2 could compensate for the functional deficiency of isoform 1.

**Figure 6:**
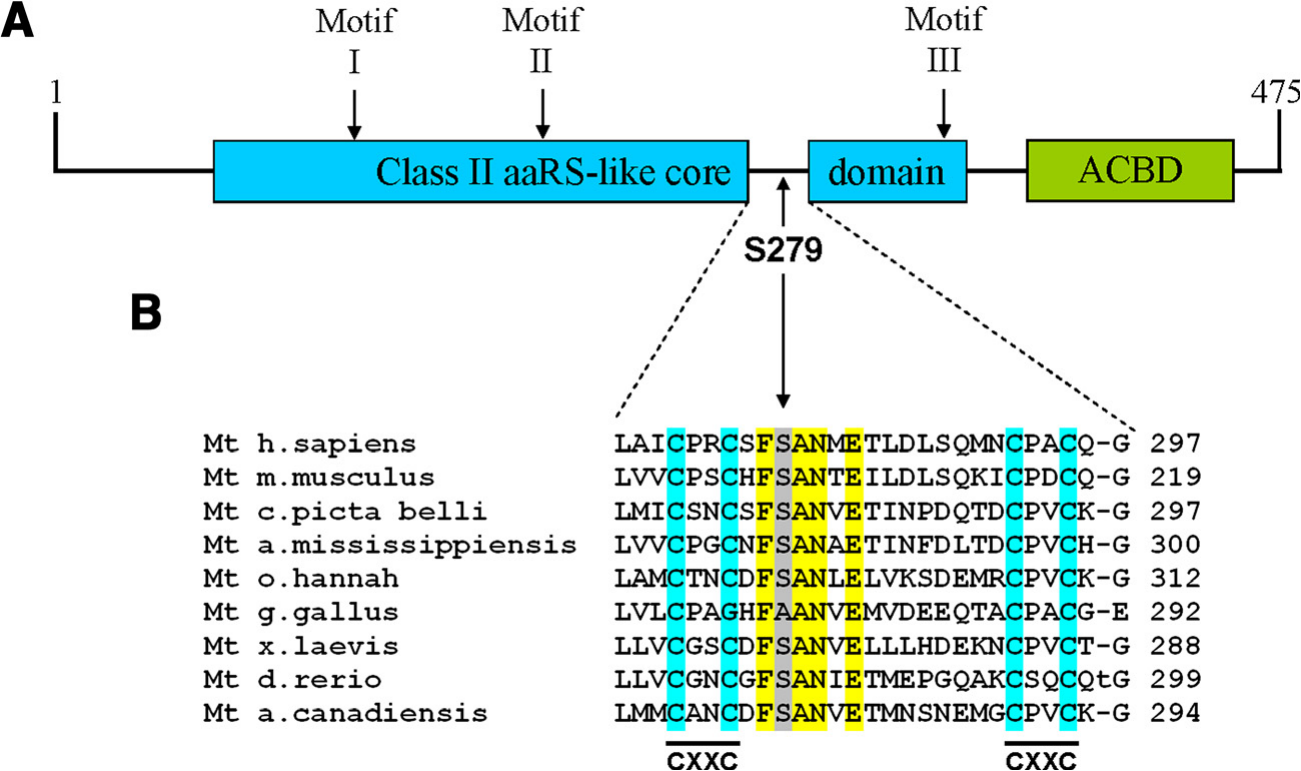
(A) Primary structure and principal domains of human *PARS2*. ACDB: anticodon-binding domain. (B) 34 residue domain specific for prolyl-tRNA synthetases. Highlighted in yellow is the conserved motif where the S279L mutation is located. This motif is flanked by CXXC motifs, a characteristic of functional domains inserted into larger proteins.

Patient II presented compound heterozygosity for two mutations in *PARS2* (NM_152268.3). Mitochondrial prolyl-tRNA synthetase also presents an anticodon-binding domain and a class II aaRS-like core domain, but their relative positions are inverted with respect to asparaginyl-tRNA synthetase (Fig.6A). The first mutation (c.1130dupC, p.K378 fs*1) is a one base insertion at codon 377 which creates a premature stop codon at position 378. This mutation is predicted to generate a truncated protein 98 residues shorter than the wild-type form. The predicted missing part contains motif 3, which is part of the active site, and part of the dimer interface. Even assuming that the truncated protein is stable, it can be predicted that the lack of such motifs totally abolishes the enzymatic activity of the protein. The second mutation (c.836C>T, p.S279L) is a missense mutation, predicted by SIFT to be damaging (although it is predicted to be benign by PolyPhen-2) and is not listed as dbSNP (build 135) nor in any of the NHLBI ESP nor HBVDB databases. S279 is located in a 34 amino acid long cluster between motifs 2 and 3 which does not belong to the class II aaRS-like core domain. A BLAST search to identify homologous clusters in other proteins showed that all matches are located in mitochondrial prolyl-tRNA synthetases (Fig.6B). Prolyl-tRNA synthetases are known in many species to mischarge alanine and cysteine. Consequently, the fidelity of aminoacylation requires an editing function, which can be located in *cis-* (an editing domain in the same prolyl-tRNA synthetase) or in *trans-* (a second protein is in charge of proofreading). These editing functions are well characterized in bacteria (Vargas-Rodriguez and Musier-Forsyth 2013) but very little is known about mischarging of amino acids by their mitochondrial counterparts. The human mitochondrial protein does not possess a full-length editing domain. Intriguingly, the p.S279L mutation is located in a highly conserved motif which shares homology with a fragment of the bacterial-type prolyl-tRNA synthetase editing domain (Fig.6B). This fragment is flanked by CXXC motifs, which is characteristic of functional domains inserted in larger proteins (Grishin 2001). From this evidence it has been postulated that mitochondrial prolyl-tRNA synthetases first acquired and then lost their *cis*-editing domain at some point during evolution (Ahel et al. 2003). It is therefore tempting to speculate that the function of the cluster in which the mutation is located is somehow related to editing, perhaps by interacting with a free-standing editing domain in *trans-*. In that case it might not be surprising that the steady state levels of prolyl-tRNS synthetase are not altered in our patient, indicating that the mutation does not have an effect in the stability of the protein, but is instead causing a purely functional deficiency.

No experimental data concerning neither the structure nor the function of the proteins encoded by human *NARS2* and *PARS2* is yet available in the literature. Therefore, all structural and functional considerations must be done in light of what is known from homologs from other species. Even though there is enough in silico evidence to propose that the mutations identified in our patients are responsible for their respective clinical phenotypes, and even if Western blotting results on patient I show a clear decrease in steady state levels of asparaginyl-tRNA synthetase, further experimental work needs to be carried out to confirm our observations.
